# An Age-Structured Extension to the Vectorial Capacity Model

**DOI:** 10.1371/journal.pone.0039479

**Published:** 2012-06-19

**Authors:** Vasiliy N. Novoseltsev, Anatoli I. Michalski, Janna A. Novoseltseva, Anatoliy I. Yashin, James R. Carey, Alicia M. Ellis

**Affiliations:** 1 Institute of Control Sciences, Moscow, Russia; 2 Duke University, Durham, North Carolina, United States of America; 3 Department of Entomology, University of California at Davis, Davis, California, United States of America; 4 Fogarty International Center, National Institutes of Health, Bethesda, Maryland, United States of America; Albert Einstein College of Medicine, United States of America

## Abstract

**Background:**

Vectorial capacity and the basic reproductive number (*R_0_*) have been instrumental in structuring thinking about vector-borne pathogen transmission and how best to prevent the diseases they cause. One of the more important simplifying assumptions of these models is age-independent vector mortality. A growing body of evidence indicates that insect vectors exhibit age-dependent mortality, which can have strong and varied affects on pathogen transmission dynamics and strategies for disease prevention.

**Methodology/Principal Findings:**

Based on survival analysis we derived new equations for vectorial capacity and *R_0_* that are valid for any pattern of age-dependent (or age–independent) vector mortality and explore the behavior of the models across various mortality patterns. The framework we present (1) lays the groundwork for an extension and refinement of the vectorial capacity paradigm by introducing an age-structured extension to the model, (2) encourages further research on the actuarial dynamics of vectors in particular and the relationship of vector mortality to pathogen transmission in general, and (3) provides a detailed quantitative basis for understanding the relative impact of reductions in vector longevity compared to other vector-borne disease prevention strategies.

**Conclusions/Significance:**

Accounting for age-dependent vector mortality in estimates of vectorial capacity and R_0_ was most important when (1) vector densities are relatively low and the pattern of mortality can determine whether pathogen transmission will persist; i.e., determines whether *R_0_* is above or below 1, (2) vector population growth rate is relatively low and there are complex interactions between birth and death that differ fundamentally from birth-death relationships with age-independent mortality, and (3) the vector exhibits complex patterns of age-dependent mortality and *R_0_*∼1. A limiting factor in the construction and evaluation of new age-dependent mortality models is the paucity of data characterizing vector mortality patterns, particularly for free ranging vectors in the field.

## Introduction

The basic reproductive number (*R_0_*) and vectorial capacity are integral parts of the language, science, and control of vector-borne disease [1]. For almost 100 years, since the initial work by Ross [2] through the contributions by Macdonald [3] and Garrett-Jones [4] into the present, deviation from simplifying assumptions of the models has rarely been examined. Important assumptions are that vectors do not senescence, vector populations are homogeneous, size of adult vector populations is constant, vector bites are delivered at random and uniformly, and infected vectors never lose their infectiousness [1]. Nowadays many basic assumptions are being revised. Because of climate change the influence of temperature and its variations on malaria transmission is investigated thoroughly [5], [6], [7]. The departure from assumption that vectors do not senescence is discussed in [8], [9]. All these considerations are important for development the intervention strategies to control vector transmitted diseases [10], [11]. In order to better understand how variation in one of the most sensitive components of a vector’s role in pathogen transmission effect transmission dynamics and estimation of *R_0_*, we explored the consequences of a shift from age-independent to more biologically realistic age-dependent vector mortality. In distinction to previous works [8], [9] we consider a set of models for vector mortality, which reflect different change of mortality with age. They are Gompertz model with exponential growth of mortality, which is traditional in demography, logistic model with mortality leveling at advanced age as in heterogeneous population, models with declining, U-shaped and hump-shaped dependencies of mortality on age. In the paper we derive the general form for vectorial capacity in aging vector population. Parameterization of the survival function 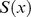 in such population gives expression for vectorial capacity for any vector mortality model and can be used for any vector control scenario.

The basic reproductive number describes the potential for spread of an infectious disease. It defines the average number of secondary cases that arise from the introduction of a single infectious individual into a completely susceptible population [12]. If *R_0_*>1 the pathogen will spread to other individuals and an epidemic can occur [2], [3]. If *R_0_*<1 transmission will not persist. For mosquito-borne disease, *R_0_* is given by the classic formula [12]:
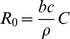
where *b* is the proportion of bites by infectious vectors that lead to host infection, *c* is the proportion of bites on infected hosts that lead to vector infection, 1/ρ is the length of time it takes to naturally clear a host from infection, and *C* is vectorial capacity, defined as the number of infective mosquito bites that arise following the introduction of a single infectious host into a susceptible population. Vectorial capacity describes the potential for a vector population to transmit a parasite [4]. It can be used to explore the relative impact of different disease prevention strategies, or put differently, ways to reduce *R_0_* below 1. For example, an effective vaccination will decrease *R_0_* by reducing infection rates of hosts (*b*) and mosquito vectors (*c*).

For two of the most important vector-borne diseases of humans, malaria and dengue [13], [14], vaccines are not yet available. Although anti-parasitic drugs are a component of malaria prevention, an analogous strategy is not planned for dengue; i.e., use of antivirals for population based dengue prophylaxis [15]. In cases like this, vector control is a prominent component of disease prevention programs. The goal is to reduce *R_0_* below 1 by reducing vectorial capacity (*C*). This can be achieved as illustrated by the classic model for vectorial capacity [12], [16]:
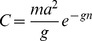
Where *m* is the number of vectors per host, *a* is the human biting rate, *g* is the mortality rate of vectors, and *n* is the extrinsic incubation period (Table 1). Many vector control programs use insecticides in an effort to decrease vectorial capacity by reducing mosquito vector population densities (*m*) and increasing the adult mosquito mortality rate (*g*). Vectorial capacity can also be used to estimate threshold density necessary for sustained transmission where *R_0_* = 1, which provides an operational target for vector control programs. If an intervention can decrease vector densities below this level, *R_0_* will be less than 1 and pathogen transmission will decrease to elimination. Although it is difficult to make precise estimates of vectorial capacity for reasons explained by Dye [17], understanding the relative impact of different intervention strategies on model components can provide helpful insights into the approaches that are most likely to successfully prevent vector-borne disease in a given situation.

**Table 1:** Parameter descriptions.

Parameter	Description
*R_0_*	Basic reproductive number, the number of secondary cases that arise from the introduction of one infected host into a completely susceptible population
*B*	Proportion of bites by infectious vectors that lead to host infection
*C*	Proportion of bites on infected hosts that lead to vector infection
1/*ρ*	Duration of viremic period in host
*C*	Vectorial capacity, number of infective bites that arise following the introduction of a single infectious host into a completely susceptible population
*M*	Number of vectors per host
*N*	Total number of mosquitoes
*A*	Biting rate
*G*	Age-independent mortality rate of vectors
*N*	Duration of extrinsic incubation period
*C_x_*	Age-specific vectorial capacity
Ω_x_	Fraction of the vector population in age class *x*
Σ	Minimum age at which vectors begin biting hosts
*Ω*	Maximum vector life span
*p_i_*	Daily survival at age *i*
*e_x_*	Remaining life expectancy at age *x*
*δ_x_*	Age distribution
*R*	Intrinsic growth rate
*S(x)*	Survival at age *x*
*g(x)*	Age-specific mortality rate of vectors at age *x*
ƒ*(x)*	Fecundity at age *x*
*H*	Entropy; describes curvature of survival curve
*Α*	Initial mortality rate
*B*	Rate of exponential mortality increase with age
*Q*	Parameter influencing location of age-dependent mortality extrema
*µ*	Parameter of age-dependence in mortality
Ξ	Parameter of age-dependence when mortality declines with age
*e_0_*	Mean lifespan
*Y(t)*	Proportion of the vector population that is infectious
*X(t)*	Proportion of the host population that is infected and infectious
*p_t_(x)*	Probability that vector of age *x* born at time *t* is infectious
ƒ_t_ *(y)*	Probability that vector born at time *t* becomes infected during 1st blood meal at age *y*
*E*	Emergence rate of vectors

Simplifying assumptions of the classic models for vectorial capacity and *R_0_* are useful approximations. Some, however, are inconsistent with mosquito biology and can constructively be viewed as starting points for more complex analyses [1]. Growing evidence indicates that age-independent vector mortality (i.e., constant *g* across all adult mosquito ages) is violated by mosquitoes in the laboratory and field [9], [18]–[24]. For example, mortality rates for the principal mosquito vector of dengue virus, *Aedes aegypti*, increase with age and, thus, are age-dependent [9], [23]–[26].

The age pattern of vector mortality is epidemiologically important because it can have strong effects on the probability that a vector will live long enough to become infectious and transmit a pathogen [17]. Horizontal transmission for pathogens, like malaria and dengue, requires that a vector is exposed to the pathogen after imbibing an infected blood meal, survives an incubation period during which the pathogen multiples or develops so that at the end of that period the vector is infectious, and then transmits the pathogen when it bites a susceptible vertebrate host. Incubation periods of many vector-borne pathogens are thought to be only slightly shorter than the mean lifespan of their vector. Relatively small changes in lifespan can, therefore, result in relatively large changes in the number of vectors that become capable of pathogen transmission. All other factors being equal, transmission rates will be highest if mortality rates decrease with vector age because the vector population will be composed of mostly older vectors that have lived long enough to become infectious. On the other hand, when vectors senesce, transmission is expected to decrease because the population age-distribution will shift to younger vectors, most of who will not live long enough to become infectious. For these reasons, understanding underlying patterns of mortality is of fundamental importance for effective design and implementation of vector control strategies. This is highlighted in the conceptual development of strategies that target the older, potentially infective portion of vector populations [27], [28]. In this paper we develop and investigated new models for *R_0_* and vectorial capacity that take age-dependence of vector mortality into account.

## Materials and Methods

### Background

Styer et. al [9] modeled total population vectorial capacity (*C_t_*) for *Ae. aegypti* as a function of age-specific vectorial capacity, *C_x_*, (i.e., daily number of potentially infective bites resulting from a mosquito of age *x* biting one infectious host) and the age structure of the population, Ω_x_:
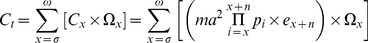
(1)where σ is the age at which mosquitoes begin biting hosts, ω is the oldest biting age class, Ω_x_ is the fraction of the mosquito population in age class *x*, *m* is the number of vectors per host, *a* is the number of host specific bites per vector per day, *n* is the duration of the extrinsic incubation period, *p_i_* is daily survival at age *i*, and *e_x+n_* is the expectation of remaining infectious life at age *x+n* (Table 1). Styer et al. [9] conducted a laboratory study to determine the pattern of age-dependent mortality for *Ae. aegypti* (i.e., exponential, logistic, or Gompertz’s mortality models) and performed simulations of equation 1 using parameter estimates for best fit mortality models. They demonstrated that *Ae. aegypti* senesce and that senescence produces different estimates of population vectorial capacity than when assuming age-independent vector mortality.

Although Styer et al.’s [9] model was useful in demonstrating how senescence can influence the dynamics of mosquito-borne diseases, it is not general enough to be easily applied to other vector species (mosquitoes other than *Ae. aegypti* as well as other non-mosquito insect vectors) with different mortality patterns. To address this issue we built on the Styer et al. [9] model in three ways. We derived (1) equations for the age structure of the mosquito population, Ω_x_ in equation 1, so that it can be used for any vector and different patterns of age-dependent mortality, (2) a new equation for *R_0_* that incorporates age-dependent vectorial capacity, and (3) a new equation for the proportion of the host population that is infectious when vector mortality is age-dependent. We illustrate the behavior of the model using parameter estimates from Styer et al. [9] for *Ae. aegypti* and different models of age-dependent mortality.

### Vectorial Capacity in Vector Population

Based on equation (1), we modeled total vectorial capacity of a population of mosquitoes, *C*. The derivation of the formula is given in Supplement S1. Some assumptions were made in the derivation. We assume that age structure of the mosquito population are constant over time and that age-dependent fecundity and mortality rates do not vary across time. The resulting expression for the vectorial capacity of a population of mosquitoes is given by:
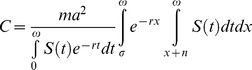
where 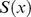 is the survival function of mosquitoes at age *x*, *r* is the intrinsic population growth rate of the mosquitoes population, and 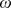 is the maximal life span. This formula gives the general form for vectorial capacity in aging vector population. It captures two important features of the population. First, it can be used for any mortality model in vector population, which produces the specific form for survival function 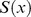. In the case of age-independent mortality survival function is given by the classical expression 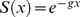, where g is mortality rate. Presented formula allows calculating vectorial capacity of a vector population in the case of any parametric or nonparametric model for age-dependent mortality. In this case 
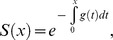
 where 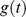 is age-dependent mortality rate. Second, the formula accounts the possibility of changing in size of the vector population. To avoid the additional hypothesis about changing in the age structure of the population we limited consideration by the case of stable population [29] where the age structure completely defined by the survival function. Table 2 summarizes formulas for vectorial capacity with age-independent and age-dependent mortality for stable (*r* and age structure are constant over time) and stationary (*r* = 0, age structure is constant over time) vector populations.

**Table 2:** Formulas for vectorial capacity in stable and stationary vector populations.

Population	Type of Mortality	Vectorial Capacity
Stable	Age-independent	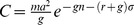
	Age-dependent	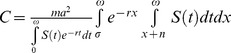
Stationary	Age-independent	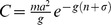
	Age-dependent	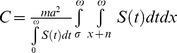

### Role of Age-dependent Mortality in Determining R_0_

Age-dependent mortality can have important implications for the spread of vector-borne pathogens through host populations. We investigated this by simulating host and vector dynamics and calculating *R_0_* for *Ae. aegypti*. A similar exercise could be done for other vector species.

Supplement S2 presents equations for the proportion of the host population that is infected or infectious when vector population exhibits age-dependent mortality. These equations were used in a species-specific example to simulate dynamics of dengue transmission in a human population by *Ae. aegypti*. We used the logistic model for age-dependent mortality:
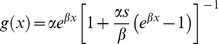
(parameters are defined in Table 1) and the parameter estimates presented in Styer et al. [9] (i.e., α = 0.0018, *β* = 0.1416, *s* = 1.0730).

To calculate the basic reproductive number, *R_0_*, in a stable vector population with age-independent mortality we used the classic expression [12]:
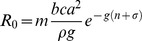
with parameters *a* = 0.75, *b = c = *0.5, *g* = 1/32, *ρ* = 0.01, *n* = 10, *σ* = 3 adopted from Styer *et al.* [9]. The general relationship between the basic reproductive number and the vector capacity is given de 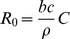 from which the basic reproductive number 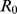
in stable population with age-dependent mortality is given by:



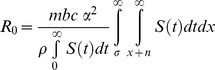
We use the same parameter values as above and the survival function for logistic mortality 
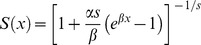
to explore the dynamics of these models.

## Results

### Vectorial Capacity for Different Patterns of Age-dependent Mortality

**Figure 1:**
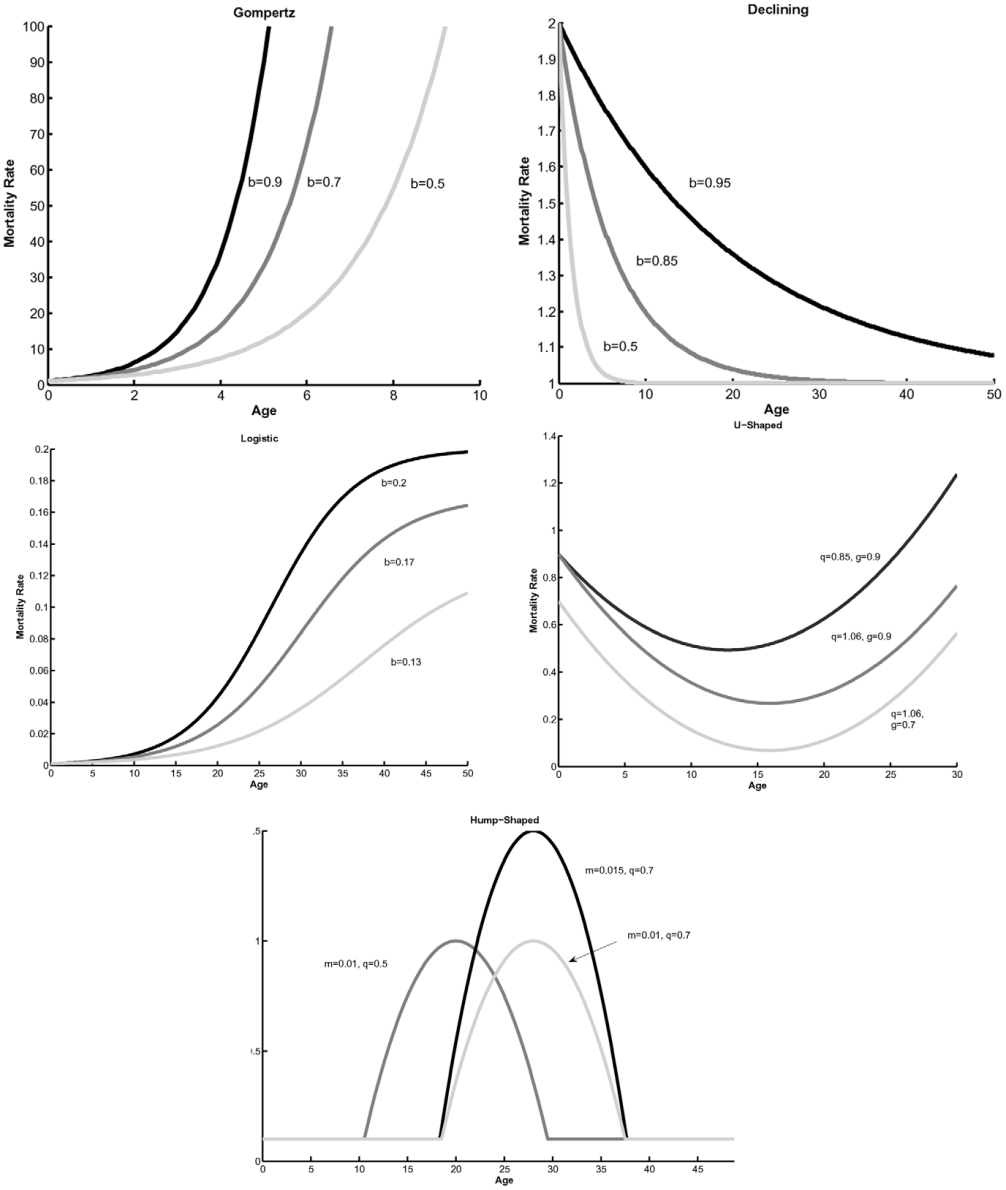
Illustration of mortality models examined with different parameter values. Parameter values not listed; Gompertz: α = 0.01, declining: µ = 0.7, *g* = 0.1, logistic: *α = *0.007, *s* = 0.2, U-Shaped: *µ* = 0.001, *e_0_* = 24, unimodal: *g* = 0.05, *e_0_* = 30.

**Figure 2:**
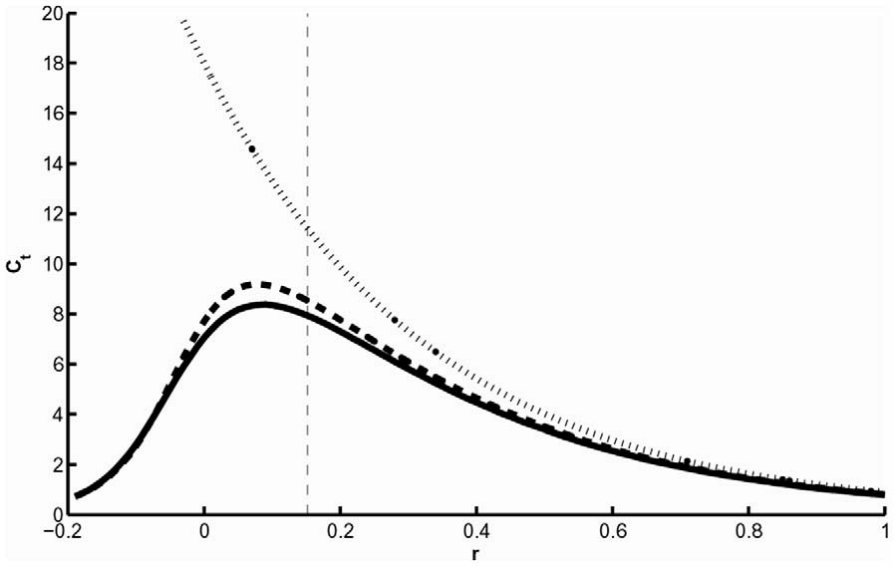
Vectorial capacity in a stable population for three mortality models (see Table 3 for functions). Parameters used in calculations are: exponential (dotted line, *g* = 0.0313), Gompertz (dashed line, α = 0.00662, *β* = 0.06234), and logistic (solid line, *α* = 0.00662, *β* = 0.06234, *s* = 1.073); taken from Styer et al. 2007a.

To examine the behavior of the model, we calculated vectorial capacity for *Ae. aegypti* as an example using parameter estimates from Styer et al. [9] for age-independent (i.e., exponential), Gompertz, and logistic mortality models (Fig. 1) with different values of intrinsic population growth, *r*. In all calculations maximal life span was set to infinity (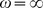). For the age-independent mortality exponential model, vector capacity is negatively related to *r* across all values of *r* examined (Fig. 2). Vectorial capacity differs most between age-independent and age-dependent mortality models at intermediate and low *r* values. If age-independent mortality increases, the corresponding vectorial capacity curve goes down and can intersect the curves for age-dependent mortalities but at any case vectorial capacity for age-independent mortality decreases with an increase of intrinsic population growth parameter *r*. When, however, the vector exhibits age-dependent mortality characterized by the logistic or Gompertz models, vectorial capacity exhibits a unimodal relationship with *r* (Fig. 2).

Next we evaluated how age-dependent mortality affects vectorial capacity of a stationary vector population (*r* = 0) with Gompertz, declining, U-shaped, and unimodal age-dependent mortality trajectories (Table 3). We did not test the logistic mortality model (Table 3) because with the parameter estimates in Styer et al. [9] it gives vectorial capacity values close to the Gompertz model (Fig. 2). Although we were primarily interested in examining the difference between the mortality models, there can be considerable variability in the form of the survival function within each mortality model. The unimodal mortality model, for example, can have variation in the age at which the maximum mortality occurs and the overall shape of that relationship.

**Table 3:** Mortality models used in this study.

Mortality Model	Function
Exponential (age-independent)	*g*(*x*) = *g*
Gompertz (increases with age)	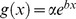
Logistic	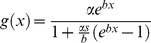
Decline with Age	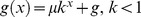
U-shaped	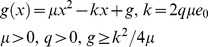
Unimodal	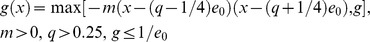

**Figure 3:**
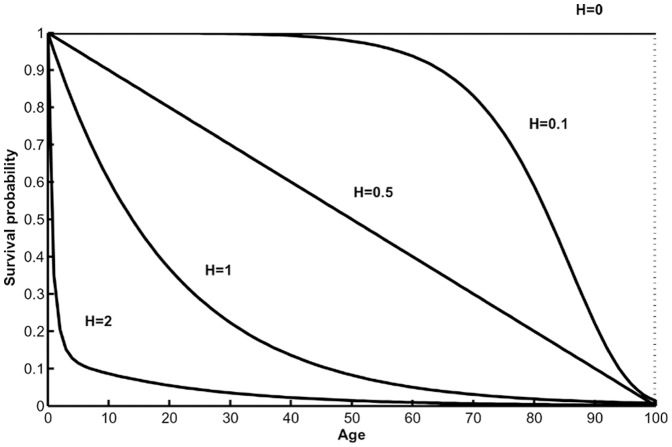
Hypothetical survivorship curves for different values of *H,* entropy.

To better examine the full range of behavior within each mortality model we present vectorial capacity as a function of life table entropy, *H* [30]. This value generalises behaviour of the survival curve and allows comparison of different curves without consideration of the corresponding parameters. Entropy describes the curvature of the survival curve (Fig. 3) and can be interpreted as the percentage change in life expectancy produced by a 1% decrease in the force of mortality at all ages [30]. Entropy is given by:
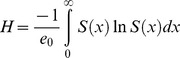
where *e*_0_ is mean life span given by 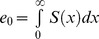 [31] and *S(x)* is survival at age *x.* It is clear that for any survival curve *H* is not negative. In the case of a non-aging population, mortality with age is constant giving a concave, exponentially decreasing survival curve (proportion surviving vs. age) and *H* = 1 (Fig. 3). For rectangular survival curves where all individuals die at exactly the same age, *H* = 0. Finally, when there is very high infant mortality followed by relatively higher survival rates for older individuals, *H*>1. Entropy is thus a continuum that represents different forms of the survival function (Fig. 3) and is directly related to the pattern of age-dependent mortality.

**Figure 4:**
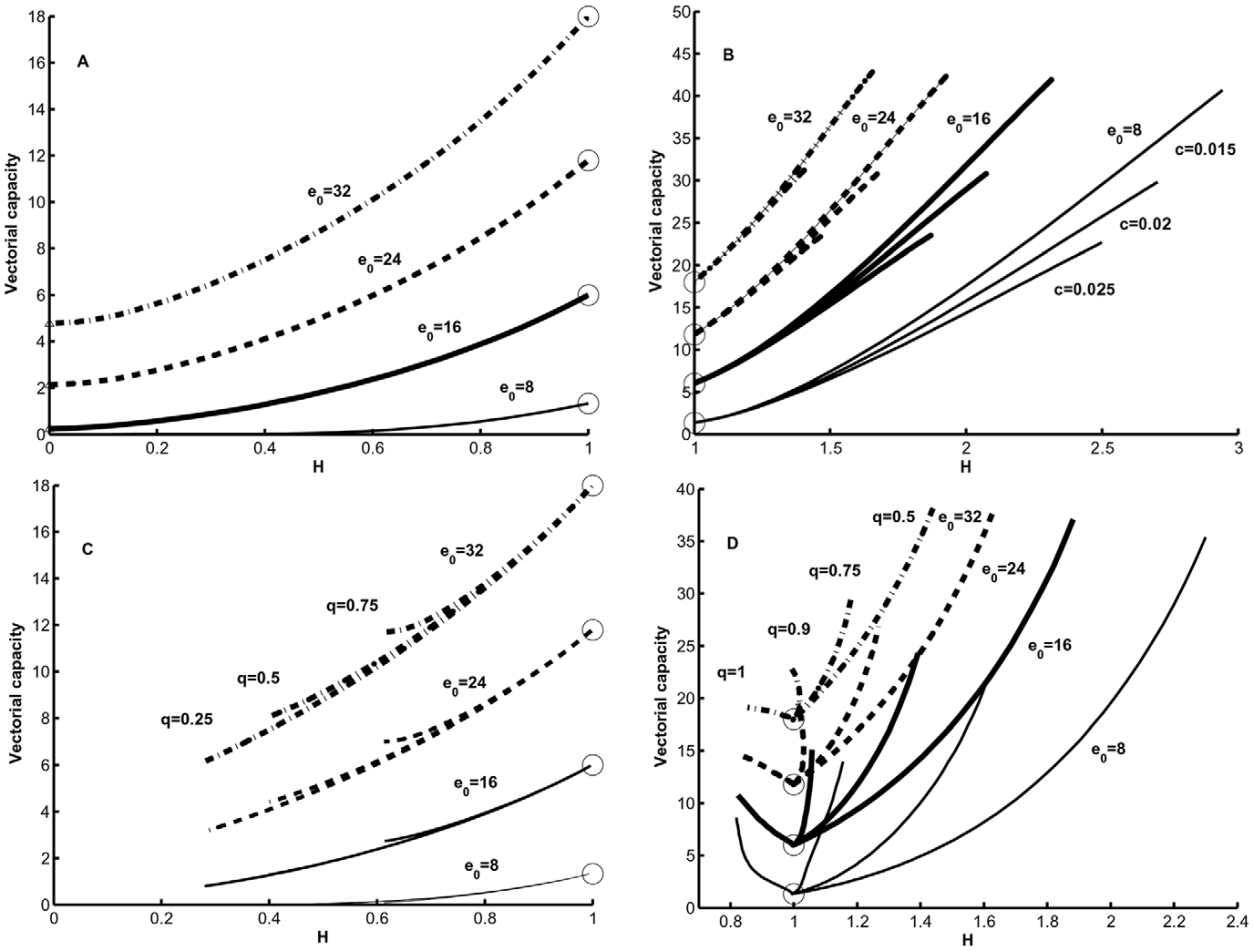
Vectorial capacity versus entropy *H* for different values of mean life span *e*_0_. (A) Gompertz’s, (B) declining, (C) U-shaped, and (D) unimodal mortality models. For each model, age-independent mortality for each life span is noted by an open circle.

Figure 4 presents vectorial capacity as function of *H* for stationary vector populations under fixed values of mean life span, *e_0_*, for different mortality models. For the Gompertz model (Fig. 4A, Table 3), age-independent mortality, and maximum vectorial capacity for a given lifespan occurs when *β*, the exponential mortality increase with age, equals 0 and *H* = 1. With a fixed value for vector lifespan, decreasing *H* and increasing *β* generates lower vectorial capacity (Fig. 4A). An increase in *β* corresponds to an increase in mortality rate across all ages (Fig. 1) or proportionally greater increase in mortality for older vectors that would have lived long enough to become infectious than younger vectors that are not old enough to have completed the pathogens incubation period (Fig. 1). For a given value of *H*, vectorial capacity decreases with decreasing lifespan. A shorter lifespan reduces the probability that a vector will become infected during its lifetime and if infected, reduces the number of times that a vector can transmit a pathogen to susceptible hosts.

For a declining mortality rate (Fig. 4B, Table 3), age-independent mortality occurs when parameter ξ = 1 (*H* = 1). In contrast to the Gompertz model, age-independent mortality gives the lowest vectorial capacity. For a given value of mean life span, vectorial capacity decreases as *H* decreases and ξ increases. In general, the decrease in vectorial capacity with increases in ξ is likely due to an overall increase in the population mortality rate (Fig. 1).

For a U-shaped relationship between vectorial capacity and age (Fig. 4C, Table 3), the minimum of the mortality function is located at *x_min_* = *qe_0_*. Changing *q* changes the location of this minimum (Fig. 1). For age-independent mortality, µ* = *0 and, as for the Gompertz model, gives the highest vectorial capacity for this model. For a given mean lifespan, vectorial capacity decreases with a decrease in *q* and *H* and an increase in *g* (Fig. 4C) because this increases overall mortality (Fig. 1).

With unimodal mortality (Table 3), the *H*-dependence of vectorial capacity differs fundamentally from the cases described above (Fig. 4D). In our calculations, we varied parameters *µ* and *g*. The location of the maximum mortality was determined as 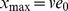. For age-independent mortality (*H = *1), vectorial capacity is at a minimum. In contrast to the case with U-shaped mortality, any deviation from age-independent mortality increases vectorial capacity. For a given lifespan, vectorial capacity can be positively or negatively related to *H* depending on the value of *q*. As *H* decreases, older vectors have a higher probability of surviving (Fig. 2). However, changes in *q* change the age at which mortality is at a maximum (Fig. 1). This is important because the age when vector mortality is at a maximum determines if mortality is relatively high or low for age groups best able to transmit a pathogen. The interaction between these 2 processes generates the complex pattern for a given lifespan.

Across all mortality models examined, mean lifespan is an important parameter determining vectorial capacity (Fig. 4). For decreasing, U-shaped, and unimodal mortality models, other parameters are also important. This is particularly true for the unimodal mortality curve where vectorial capacity is sensitive to changes in the parameter determining the location of the mortality maximum.

### Role of Intrinsic Growth Rate r

Figure 2 shows vectorial capacity in a stable population in dependence on intrinsic rate of grows *r* for three mortality models: exponential, Gompertz and logistic. The largest differences in vectorial capacity between these models occurs when value of *r* is intermediate or low. For age-independent mortality, lower *r* values correspond to lower birth rates because the mortality rate is constant. In this case, decreasing *r* by decreasing birth rates causes an increase in the proportion of the vector population in older age classes that are capable of transmitting a pathogen, which in turn increases vectorial capacity. Conversely, with age-dependent mortality age-specific mortality and fecundity rates vary non-linearly with different values of *r*. The complex interaction between nonlinear birth and death rates and *r* lead to a decrease in vectorial capacity as *r* decreases, which can occur because mean age and thus transmission potential decrease as *r* decreases. This reinforces the need for new data that can be used to refine and test predictions regarding associations between intrinsic rate of growth and the complexity of age-dependent mortality.

### R_0_ and Dynamics of Host Infection

**Figure 5:**
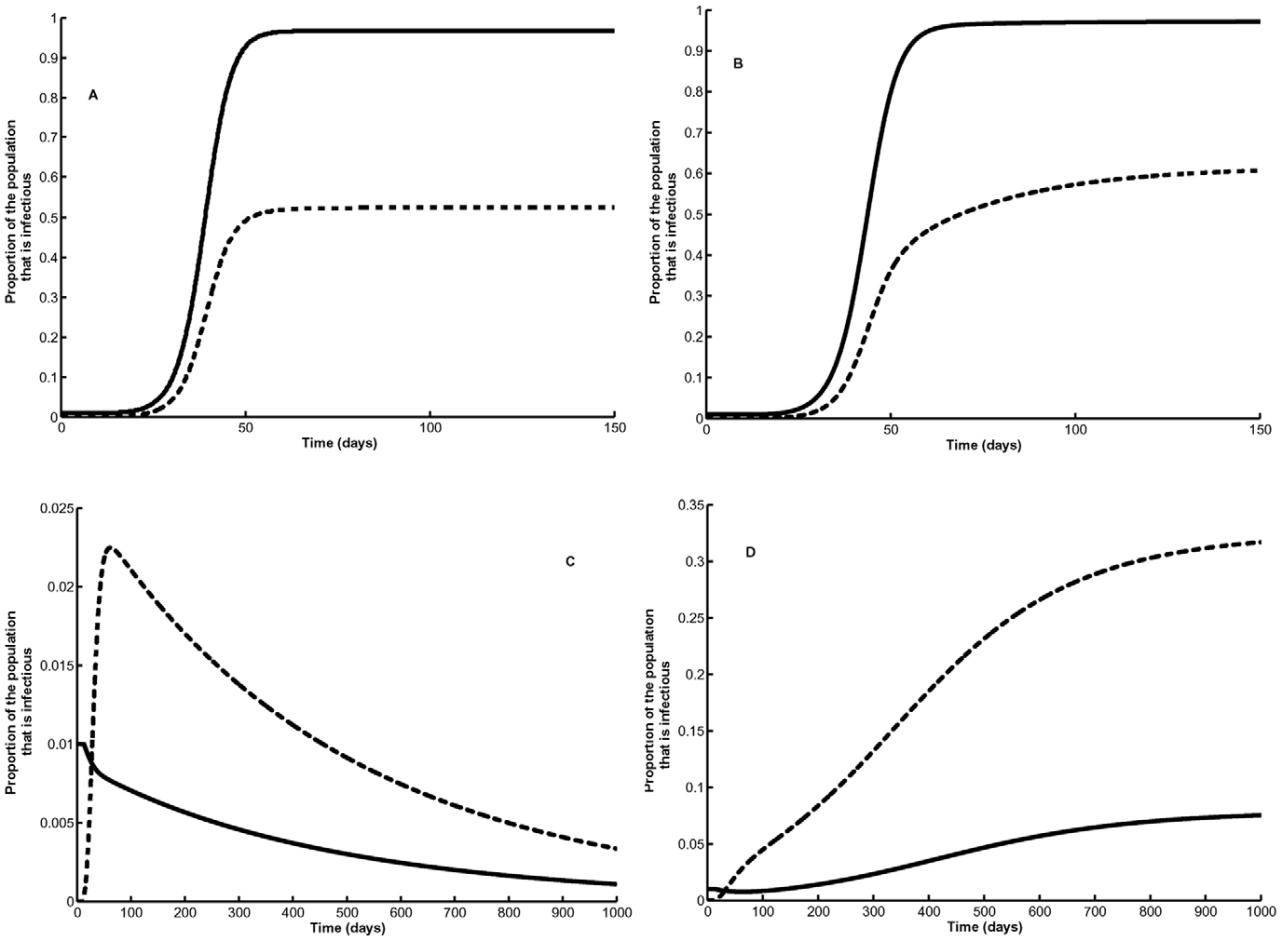
Transmission dynamics when an infectious host is introduced at time = 0. (A) age-dependent mortality, *m = *1.5, (B) age-independent mortality *m* = 1.5, (C) age-dependent mortality, *m = *0.007, (D) age-independent mortality *m* = 0.007. Solid line denotes humans and dashed line mosquitoes.

Depending on mosquito population density, there are epidemiological important differences in dynamics of human and mosquito infections with age-dependent versus age-independent vector mortality (Fig. 5). When mosquito density is relatively high (*m = *1.5), using the logistic mortality model and parameter estimates obtained by Styer et al.’s [9] mortality study, the basic reproductive number with age-dependent mortality is 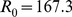 and 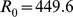 for the age-independent mortality. For such high values of *R_o_*, epidemic levels of transmission would be expected for both age-independent and -dependent mortality and stationary values would be similar for infectious host and vector populations (Fig. 5A,B). A different type of dynamics occurs when using the same parameter estimates, but with a low mosquito density, *m* = 0.007. With age-dependent mortality 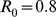 and the proportion of infected vector and host populations declines with time (Fig. 5C,D). For age-independent mortality_
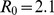
_ and the infected proportion of the population increases over time.

## Discussion

The new mathematical models derived and investigated in this paper extend the theoretical and empirical understanding of age-dependent vector mortality in ways that can add to the conceptual basis of vector-borne disease prevention. The formula for dependence of vectorial capacity from the vector survival is general enough and can be used in calculation of vectorial capacity for any of age-dependent mortality of vectors. One immediate utility of the formula is in the case, when a vector is exposed to two causes of death: exogenous, which does not depend on the vector age, such as predation, swatting, weather conditions, and endogenous, related to ageing and vector senescence. It is realistic to consider these two causes as independent hazards, which mathematically means that the resulting mortality is a sum of the exogenous and endogenous mortalities. The presented formula allows calculations of vectorial capacity to find the limits of control, when measures, aimed to increase the exogenous mortality, can be effective against the background of endogenous age-dependent mortality. In this case the value of age-independent mortality component can be considered as a parameter for construction of optimal control strategy under given resource limitations.

Introduction of age-dependent mortality broadens and refines the vectorial capacity paradigm by introducing an age-structured extension to the model. It encourages further research on the actuarial dynamics of vector populations and the contribution of dynamics in vector mortality to dynamics in pathogen transmission. It provides a quantitative basis for understanding the relative impact of reductions in vector longevity compared to other strategies for prevention of vector-borne disease; i.e., various forms of vector control, vaccines, and anti-pathogen drugs that are applied separately or in combination.

Our analysis indicates that different age-dependent patterns of mortality can influence vectorial capacity differently. Although mean lifespan remains an important determinant of vectorial capacity, other parameters that affect the shape of a mortality curve are also important. We used demographic entropy to illustrate such influence in one value, which is free from the specific parameterization of age-dependent mortality. In addition to mean life span, our analysis of entropy illustrates how survival curves can differ from the original assumption of exponential, or age-independent, mortality profile.

The shift from age-independent to age-dependent mortality can be viewed as conceptually advantageous because it captures transmission dynamics in a more biological relevant way [9]. It also increases the mathematical complexity of the models, which raises questions about their general applicability in applied, epidemiologic contexts. A key issue in this regard concerns the strength of the insights gained from the increased complexity, biological and mathematical. That is, does including the additional age-dependent elements substantially improve the power of the models for guiding disease surveillance and prevention? Results from our analyses indicate that there are important differences in the epidemiologic output from age-dependent vs -independent vector mortality models. Understanding the nature of output differences is challenging because it depends on variation in complex characteristics of the system being examined. Examples include novel vector control strategies that target older vectors or aim to shorten vector lifespan [27], [32] or prevent disease by pathogen elimination [33].

Our vectorial capacity model has several limitations. It assumes no emigration or immigration of vectors or hosts, which can be particularly important if rates are not equal. It is deterministic and does not account for individual variatiability (hidden heterogeneity) in chances of survival. In some cases it may be difficult to fit simple mortality functions (e.g., Gompertz, Weibull, etc. [30]) to field or laboratory data [34]. More research is needed to examine the impact on vectorial capacity when vector age-structure and population growth rate vary over time.

Estimates of vectorial capacity, and thus *R_0_*, for mosquito-borne pathogens differ most between cases of age-independent and age-dependent mortality when: (1) vector densities are relatively low, (2) vector population growth rate is relatively low and there are differences in the complex interactions between birth and death; and (3) vectors exhibit complex patterns of age-dependent mortality. In many parts of the world vector control is an integral or even primary component of vector-borne disease prevention. Our analyses indicate that when vector control programs are successful and mosquito densities are reduced to low levels, the mortality model used to predict sustainability of that success or the effort needed for the final push to eliminate the pathogen can lead to strikingly different conclusions. At the same low vector density (*m* = 0.007), age-independent mortality predicts increasing, epidemic transmission (*R_o_*>1) and age-dependent mortality predicts local pathogen extinction (*R_o_*<1). An age-independent model may overestimate the effort needed to meet public health goals at low vector densities.

Our conclusions support earlier results indicating that age-dependent vector mortality can influence transmission dynamics and the success of disease prevention strategies in meaningful ways [9], [27], [35]. In a similar study, Bellan [8] demonstrated that age-dependent vector mortality has important effects on vectorial capacity and vector control. By focusing only on the logistic mortality model and possible effects of two control measures (decreased survival and decreased recruitment that is equal across all ages, with each intervention affecting a single parameter) and simplifying the equation for vectorial capacity, he demonstrated that the effects of interventions may be over- or under-estimated when assuming age-independent survival. Our study, however, goes further by providing a single complete formula for vectorial capacity that can be used for any vector, mortality model, and control scenario. Our formula allows researchers to calculate vectorial capacity and assess the effects of various control measures for their own particular system. Importantly, this equation may be easily expanded to include more complex functions affecting vectorial capacity including density-dependent mortality (in addition to age-dependent mortality) or factors affecting biting rate.

It is important to note that despite growing evidence showing notable effects of age-dependent mortality on estimates of vectorial capacity and effects of control, we still know very little about the prevalence and pattern of age-dependent mortality in natural mosquito populations [20], [23], [24], [25]. Age-dependent mortality and the diversity of its patterns has been examined for non-vector insects [20], [34]. Most of those studies lacked sufficiently large sample sizes to accurately estimate mortality rates of relatively rare, older-aged individuals [34]. We similarly know little about what factors govern disease vector mortality patterns or how mortality patterns vary through space and time. Theoretically, variation in patterns of age-dependent mortality could cause frequent and dramatic fluctuations in vectorial capacity and entomological thresholds below which epidemic pathogen transmission will cease. New techniques to better estimate patterns of age-dependent vector mortality, how mortality patterns vary in space and time, and the factors determining those patterns are needed to better understand when and how age-dependent vector mortality has its greatest affects on transmission dynamics and disease intervention campaigns.

The field of aging research can make substantial contributions to improved understanding and more efficient prevention of vector-borne disease because it deals with factors and mechanisms affecting age-specific patterns of mortality among different species [36], [37]. Identifying concepts and interventions capable of accelerating vector aging processes, and understanding how such manipulations affect pathogen transmission parameters can stimulate investigation of new approaches for vector-borne disease control. Consideration of more realistic situations will require more sophisticated models and more comprehensive computational analyses of alternative scenarios. For example, undefined heterogeneities in vector mortality patterns are likely to be important determinants in the success or failure of vector control programs. Due to variation within and between mortality patterns, a strategy that works well at one place and time may not work at another. Our analysis indicates that a more sophisticated analytical framework, which is mathematically and computationally plausible, will stimulate increasingly insightful thinking about age-dependent vector mortality and prevention of vector-borne disease. Although our analyses use data from a single mosquito species, *Ae, aegypti*, our models are intended to have broad application for a wide range of vector species and vector-borne diseases.

## Supporting Information

Supplement S1 **Vectorial capacity in stable and stationary vector population.**
(DOC)

Supplement S2 **Proportion of the host population that is infected or infectious when the vector population exhibits age-dependent mortality.**
(DOC)
