# Supplementary material for: An Age-Structured Extension to the Vectorial Capacity Model
Source: PLoS One. 2012 Jun 19;7(6):e39479. doi: 10.1371/journal.pone.0039479 (PMC3378582; doi:10.1371/journal.pone.0039479)
Supplement: Supplement S1 — Vectorial capacity in stable and stationary vector population. (DOC) [file pone.0039479.s001.doc]

**Supplement S1**

*Vectorial capacity in stable and stationary vector population*

Let $q\left(x\right)$ denotes the age distribution in a population of mosquitoes. The fraction of the mosquito population in age interval $\left[x,x+dx\right]$ for small $dx$ is close to $q\left(x\right)dx$ and daily number of potentially infective bites resulting from mosquitoes of all ages in such population, called vectorial capacity $C$, is given by:

$C=\int_{\sigma}^{\omega} C_{x}q(x)dx$. (SI.1)

$C_{x}$ denotes age-dependent vectorial capacity, which is the mean number of potentially infective bites that one mosquito will produce for the rest of its life under the condition that the first bite on an infectious host was made at age *x*. In the case of age-independent mortality, *C_x_* does not depend on age *x* and is calculated by the classical formula [16]

$C=\frac{ma^{2}}{g}e^{-gn}$,

where *m* – number of vectors per host, *a* – human biting rate, *n* – duration of intrinsic incubation period, and *g* – age-independent mortality rate of vectors. If mosquito mortality changes with age one should calculate the rate of infective bites $C_{x}^{t}$ at age *t* under the condition that the first bite was on an infectious host at age *x*. This is the product of the rate for a mosquito at age *x* to bite an infectious host and the rate for the same mosquito to bite a host at age *t*. This gives an expression$C_{x}^{t}=a^{2}S\left(t|x\right)$, where $S\left(t|x\right)$ is the probability of vector survival to age *t* given survival to age *x*. The infectious mosquito can infect a host only when the extrinsic incubation period ends after age *x+n*. This means that in order to obtain age-dependent vectorial capacity $C_{x}$ requires integration of $C_{x}^{t}$ on *t* from *x+n* through the maximum life span $\omega$. Considering that $S\left(t|x\right)=S\left(t|x+n\right)S\left(x+n|x\right)$ leads to the following expression

$$\begin{matrix} C_{x} =\int_{x+n}^{\omega} C_{x}^{t}dt=\int_{x+n}^{\omega} a^{2}S\left(t|x\right)dt \\ =\int_{x+n}^{\omega} a^{2}S\left(t|x+n\right)S\left(x+n|x\right)dt \\ =a^{2}S\left(x+n|x\right)\int_{x+n}^{\omega} S\left(t|x+n\right)dt. \end{matrix}$$

The last integral equals remaining life expectancy at age *x+n* $e_{x+n}=\int_{x+n}^{\omega} S\left(t|x+n\right)dt$. By substitution we obtain the final expression

$C_{x}=a^{2}S\left(x+n|x\right)e_{x+n}$. (SI.2)

It is clear, that without aging and $\omega=\infty$ we have $S\left(x+n|x\right)=e^{-gn}$ and $e_{x+n}=\frac{1}{g}$ which leads to the classical formula for vectorial capacity

$$C=\int_{\sigma}^{\omega} \frac{a^{2}}{g}e^{-gn}q\left(x\right)dx=\frac{a^{2}}{g}e^{-gn}\int_{\sigma}^{\omega} q\left(x\right)dx=m\frac{a^{2}}{g}e^{-gn}$$

To obtain the expression for the survival function $S\left(x+n|x\right)$ for an aging mosquito we assume that the mosquito population is stable (i.e., the intrinsic population growth rate, r, and age structure are constant over time) and that age-dependent fecundity and mortality rates do not vary across time. The age distribution in such populations can be represented as [29]:

$q\left(x\right)=\frac{S\left(x\right)e^{-rx}}{\int_{0}^{\omega} S\left(t\right)e^{-rt}dt}=\frac{\exp\left(-\int_{0}^{x} g(u)du\right)e^{-rx}}{\int_{0}^{\omega} \exp\left(-\int_{0}^{t} g(u)du\right)e^{-rt}dt}$ (SI.3)

where *g*(*u*) is mortality rate at age *u*, and *r* is the intrinsic population growth rate defined as the solution of the Lotka equation:

$\int_{0}^{\omega} f\left(x\right)\exp\left(-rx-\int_{0}^{x} g\left(u\right)du\right)dx=1$.

In the last equation $f\left(x\right)$is fecundity at age *x*.

Substituting equations (SI.2) and (SI.3) into equation (SI.1) and assuming that the biting rate, *a*, is constant across age and time gives:

$$C=\frac{ma^{2}}{\int_{0}^{\omega} S\left(t\right)e^{-rt}dt}\int_{\sigma}^{\omega} S\left(x\right)S\left(x+n|x\right)e^{-rx}\int_{x+n}^{\omega} S\left(t|x+n\right)dtdx$$

$C=\frac{ma^{2}}{\int_{0}^{\omega} S\left(t\right)e^{-rt}dt}\int_{\sigma}^{\omega} e^{-rx}\int_{x+n}^{\omega} S\left(t\right)dtdx$ (SI.4)

Equation (SI.4) is a complete formula for vectorial capacity, which is valid for any pattern of age-dependent (or independent) mortality and any vector.

In a case if the intrinsic population growth rate *r* is positive the number of vectors per host *m* will grow exponentially with time and vectorial capacity of such population will row exponential as well. Negative value of *r* corresponds to exponential decrease of *m* and eradication of vector population. In the case of stationary population of vectors *r=*0 and the number of vectors per host *m* does not change with time.
