# Supplementary material for: An Age-Structured Extension to the Vectorial Capacity Model
Source: PLoS One. 2012 Jun 19;7(6):e39479. doi: 10.1371/journal.pone.0039479 (PMC3378582; doi:10.1371/journal.pone.0039479)
Supplement: Supplement S2 — Proportion of the host population that is infected or infectious when the vector population exhibits age-dependent mortality. (DOC) [file pone.0039479.s002.doc]

**Supplement S2**

*Proportion of the host population that is infected or infectious when the vector population exhibits age-dependent mortality*

Denote by *X(t)* the proportion of the host population that is infected and infectious at time *t* and by *Y(t)* the proportion of the vector population that is infected at time *t*. In [16] the change in *X(t)* with age-independent mortality was modeled as:

$\frac{d}{dt}X(t)=mba\left(t\right)Y(t)(1-X(t))-pX(t)$,

where *m* is number of vectors per host, *b* is the proportion of bites by infectious vectors that lead to infection in the host, *a* is the human biting rate of the vector, 1/*p* is the duration of host viremic period. Age-dependent mortality can be incorporated into this equation by modifying the proportion of the vector population that is infectious at any given time *Y*(*t*).

Let $p{}_{t}\left(x\right)$ be the probability that a vector of age *x* born at time *t* is infectious. Define *f_t_*(*y*) as the probability that a vector born at time *t* becomes infected during their first blood meal at age *y*. The probability of surviving to age *x* and being alive at age *y*+*n* is $S\left(x|y+n\right)$ and the probability of surviving the extrinsic incubation period *n* after a blood meal at age *y* is$S\left(y+n|y\right)$. These functions are interrelated by the following equation

$p_{y}(x)=\int_{\sigma}^{x-n} f_{y}\left(t\right)S\left(t+n|t\right)S\left(x|t+n\right)dt$. (SII.1)

The probability that a vector born at time *t* becomes infected during their first blood meal at age *y*, *f_y_*(*t*), is itself a function of the proportion of infected hosts in the population, survival to age *y*, *S(y)*, the proportion of bites on infected hosts that lead to infection in a vector, *c*, and the biting rate of vectors at age *y*, *a(y)*. Here, we assume that the biting rate and survival (*a*, *S*, respectively) vary with age (*y*), but that those age-dependent values do not change through time. This gives:

$f_{y}\left(t\right)dt=ca(t)X\left(y+t\right)e^{-c\int_{\sigma}^{t} a(\tau)X\left(y+\tau\right)d\tau}S(t)dt$. (SII.2)

Substituting equation (SII.2) into (SII.1) gives:

$$p_{y}(x)=cS(x)\int_{\sigma}^{x-n} a(t)X(y+t)e^{-c\int_{\sigma}^{t} a(\tau)X(y+\tau)d\tau}dt$$

The last equation can then be used to model the proportion of the vector population that is infected at a given point in time (*Y(t)*):

$$\begin{matrix} Y(t)=\frac{1}{N\left(t\right)}\int_{\sigma+n}^{\infty} \varepsilon\left(t-x\right)p_{t-x}(x)dx \\ =\frac{1}{N\left(t\right)}c\int_{\sigma+n}^{\infty} \varepsilon\left(t-x\right)S(x)\int_{\sigma}^{x-n} \alpha(\xi)X(t-x+\xi)e^{-c\int_{\sigma}^{t} \alpha(\tau)X(t-x+\tau)d\tau}d\xi dx, \end{matrix}$$

whereis the number of vectors at time *t*, is the number of vectors that emerged during the time interval$\left(t,t+dt\right]$*.* In a stationary population, the total number of vectors (*N*) and the rate of vector emergence ($\varepsilon$) do not depend on time which leads to the relationship$N=\varepsilon\int_{0}^{\infty} S(\tau)d\tau$.
